# Is There a Better Biomaterial for Dental Implants than Titanium?—A Review and Meta-Study Analysis

**DOI:** 10.3390/jfb13020046

**Published:** 2022-04-20

**Authors:** Håvard J. Haugen, Hongyu Chen

**Affiliations:** 1 Department of Biomaterials, Institute of Clinical Dentistry, University of Oslo, 0318 Oslo, Norway; 2 Department of Biomedical Engineering, Rensselaer Polytechnic Institute, Troy, NY 12180, USA; chenhongyu426@gmail.com

**Keywords:** meta-study analysis, dental implant, peri-implantitis, Randomized Controlled Trials (RCTs), abutments, surface modification, clinical outcome, zirconia

## Abstract

This article focuses on preclinical studies and reviews the available evidence from the literature on dental implant and abutment materials in the last decade. Specifically, different peri-implantitis materials and how surface modifications may affect the peri-implant soft-tissue seal and subsequently delay or hinder peri-implantitis are examined. This review analyzed more than 30 studies that were Randomized Controlled Trials (RCTs), Controlled Clinical Trials (CCTs), or prospective case series (CS) with at least six months of follow-up. Meta-analyses were performed to make a comparison between different implant materials (titanium vs. zirconia), including impact on bone changes, probing depth, plaque levels, and peri-implant mucosal inflammation, as well as how the properties of the implant material and surface modifications would affect the peri-implant soft-tissue seal and peri-implant health conditions. However, there was no clear evidence regarding whether titanium is better than other implant materials. Clinical evidence suggests no difference between different implant materials in peri-implant bone stability. The metal analysis offered a statistically significant advantage of zirconia implants over titanium regarding developing a favorable response to the alveolar bone.

## 1. Introduction

Tooth loss usually occurs in patients who suffer from oral diseases and traumas. It impairs masticatory function and may also cause gradual resorption of the alveolar bone [1]. Removable prostheses were conventionally used to restore functional defects and improve esthetics without teeth. In comparison, dental implants possess various advantages over unfixed dentures, such as preservation of adjacent teeth and long-term success. With the continuous optimization of dental implants and improvement of clinical techniques, the survival rate of implants has been reported to be up to 90–95% over periods of 5–10 years [2]. Dental implants have become a popular alternative to conventional prostheses during the past decade. However, implant failures do occur due to different factors such as post-operative infections and occlusal overload. The inflammatory process at the peri-implant bone region, namely peri-implantitis, is one of the significant concerns jeopardizing the long-term efficacy of implants. Peri-implantitis is a destructive inflammatory process affecting the soft and hard tissues surrounding dental implants [3]. Peri-implantitis involves severe complications at the implant site featured by the presence of pus, bleeding upon probing, deep pocket, and bone resorption [4]. Without successful treatment, peri-implantitis could lead to severe destruction of the supporting bone and eventual implant loss and is becoming a serious challenge in implantology [3].

Peri-implant tissue has been well discussed in terms of healing and clinical outcomes, showing a clear benefit for the patients’ masticatory efficiency and quality of life [5]. The changes in the surface morphology have shortened the treatment times and brought about an earlier delivery of functional restorations [6], which has enabled the clinicians to provide adequate treatment in the vast majority of cases. Early clinical publications indicated that peri-implantitis occurred in the first year of loading. Numerous attempts have been made to minimize or eliminate such bone loss. Despite this, the timing and the reason for peri-implantitis are not always obvious [3,7].

However, the current literature intensely focuses on and is limited to the clinical reports comparing the different treatment options for the current patient population. Even though clinical data are highly suitable for treatment providers, patients, and quality assurance, experimental research in the preclinical stage should not be neglected because of the novel ideas that might result in new treatment strategies in the future. For advancing the art and science in dentistry, such studies could be considered more significant than the clinical studies applying the concepts and products already available today if following sound and scientific principles.

This article focuses on preclinical studies, a stage of research that begins before testing in humans, typically in laboratory animals, and reviews the available evidence from the literature in the last decade on dental implant materials to combat/prevent peri-implantitis. Specifically, different materials and how surface modifications may affect the peri-implant soft-tissue seal and subsequently delay or hinder peri-implantitis are examined. This review analyzed more than 30 studies that were Randomized Controlled Trials (RCTs), Controlled Clinical Trials (CCTs), or prospective case series (CS) with at least six months of follow-up. Meta-analyses were performed to make a comparison between different implant materials (titanium vs. zirconia), including impact on bone changes, probing depth, plaque levels, and peri-implant mucosal inflammation, as well as how the properties of the implant material and surface modifications would affect the peri-implant soft-tissue seal and peri-implant health conditions. This review intends to provide room for new concepts, new ideas, and new products that will help to address our patients’ future needs.

## 2. Literature Search

An extensive literature review was conducted in the last ten years to examine the kind of implant needed to combat peri-implantitis and how surface modification may affect the peri-implant soft-tissue seal and subsequently delay or hinder peri-implantitis. The review examined over 100 studies. The studies were identified by examining several electronic databases, such as PubMed and the Cochrane Central Register of Controlled Trials. The following keywords were used for the searches: dental implant AND Abutments AND zirconia AND alloy AND surface treatments. The search was restricted to articles published in the English Language from 2004 to 2020. Next, journals such as Clinical Oral Implant Research, The Journal of Clinical Periodontology, International Journal of Oral & Maxillofacial Implants, International Journal of Periodontics and Restorative Dentistry, International Journal of Prosthodontics, Journal of Periodontology, Journal of Prosthetic Dentistry, Implant Dentistry, Journal of Oral Implantology, Journal of Periodontal Research, and Clinical Implant Dentistry and Related Research were searched for additional articles for the years 2004–2020.

The review also examined over 30 studies for the meta-analysis. A meta-analysis is a statistical analysis that combines the results of multiple scientific studies. For the searches, the following keywords were used: dental implant, abutments; implant, biological width, peri-implant soft tissues, bone changes, probing depth, plaque levels, and peri-implant mucosal inflammation. Studies included were Randomized Controlled Trials (RCTs), Controlled Clinical Trials (CCTs), or prospective case series (CS) with at least six months of follow-up, and meta-analyses were performed to compare materials (titanium vs. zirconia) and to evaluate the impact of various implant materials on bone changes, probing depth, plaque levels, and peri-implant mucosal inflammation. All the publications were grouped based on the study’s nature—animal studies, human histologic analysis, clinical trials, in vitro studies, the in vivo, and traditional reviews. Several set criteria were used depending on the study’s nature to determine the studies to be included in the meta-analysis. The following inclusion criteria were adopted for the animal studies: (1) the number and type of tested animals need to be mentioned clearly; (2) the sample size of test animals needs to be less than 4 in each treatment category, and (3) the test and control groups need to be included. The clinical studies were included if they reported a pronounced effect on the experiment, the control group for titanium implant or a piece implant, and at least six months of follow-up analysis. The results were considered explicit if the study reported a soft-tissue seal.

## 3. Dental Implants

Dental implants have become widely accepted and implemented in the last decade to replace missing teeth and support fixed and partially removable prostheses. Despite overall high long-term survival rates (96.1% after ten years and 83.8% after 25 years) and intensive periodontal and prosthetic maintenance, implant failures can still occur. In the past decade, substantially increased evidence has been provided to indicate that bacterial biofilm-induced peri-implant inflammation could affect both soft and hard tissues, which have eventually brought about implant failure. This inflammatory condition is distinguished as peri-implant mucositis and peri-implantitis.

Peri-implantitis was first described by Mombelli et al., as an infectious disease with several common characteristics with periodontitis [8,9]. From the clinical perspective, there was a lack of consensus on a clear definition for peri-implantitis due to complex etiological and clinical factors associated and was often case-by-case. Specifically, there was confusion between peri-implantitis and peri-implant mucositis: the former was mainly defined as an inflammatory response of the peri-implant mucosa with marginal bone loss, while the latter was focused on soft tissue inflammation. To begin with, Berglundh et al. [10] defined peri-implantitis as a plaque-related inflammatory condition that occurs around the dental implant(s). At the same time, peri-implant mucositis refers to inflammation in the adjacent gingival tissues and has no signs of loss of supportive bones after the first placement. The primary symptom of inflammation associated with peri-implantitis and peri-implant mucositis is bleeding on probing. On the other hand, inflammation of the mucosa that surrounds the dental implant(s) and progressive loss of bone tend to be the main clinical signs of both peri-implant mucositis and peri-implantitis [10].

In the recent past, peri-implant diseases (peri-implantitis and mucositis) have been extensively researched, and different treatments for these diseases were discussed. Specifically, the selection of implant materials and various material surface modifications has been studied to improve treatment outcomes and prevent inflammatory conditions that are usually associated. The material used to fabricate dental implants is critical in providing ideal mechanical properties, such as stiffness and tensile strength, and preventing the onset of inflammation surrounding the implant. Among various options, titanium (Ti), alloys, and ceramic-based zirconia (ZrO_2_) have been examined with the highest efficacy to manage inflammation and prevent peri-implant diseases.

Another critical aspect is material surface modifications, such as chemical and biological treatment of the material surface. Implants with surface modifications would prefer roughness and prevent inflammatory cell recruitment. Figuero et al. [11] examined the interventions used to manage peri-implantitis and peri-implant mucositis. Based on controlled clinical trials, it was found that the utilization of antiseptics, antibiotics, and mechanical debridement of the implant surfaces by curettes, air-abrasive devices, lasers, and ultrasonic devices could help to treat peri-implantitis and peri-implant mucositis. These interventions decreased the probing pocket depth and bleeding and inflammation of the lesions [11].

Similarly, Chala et al. [12] examined the efficacy of using lasers in treating peri-implantitis and peri-implant mucositis. Based on the evaluation of findings from nine Randomized Controlled Trials, Chala et al. found that adjunct laser application, a non-surgical therapy/treatment, had a significant impact on the treatment of peri-implantitis and peri-implant mucositis over a shorter-term period. The authors also recommended surgical intervention when a non-surgical approach is not clinically significant/practical [12]. In the following sections, implant materials and promising surface modification methods will be introduced and discussed.

## 4. Dental Implant Materials

### 4.1. Titanium

Titanium, a lustrous transition metal with atomic number 22, is widely used to manufacture dental implants [13]. Its biocompatibility due to inert behavior in the living tissue was already documented in 1951 by Gottlieb Leventhal [14]. Bengt Kasemo further elaborated on the biocompatibility of titanium and connected its superior properties as an implant material to the 2–10-nm-thick oxide layer, which instantly formed on titanium in the presence of oxygen [15]. Due to this oxide layer, titanium features high polarization resistance, protecting the metal against corrosion, hindering the release of metallic ions into the human body [16,17,18]. As a result of titanium oxide’s high dielectric constant, the surface oxide film was an attractive site for establishing chemical bindings and the attachment of a large spectrum of biomolecules [19,20]. The bioactivity, osseointegration, and biocompatibility features of titanium play an essential role in promoting bone formation in direct contact with the metal surface after dental implant placement; therefore, titanium dental implants have shown an excellent survival rate and effectiveness [21,22]. In addition, titanium promotes osseointegration, which is crucial in the success of the dental implant material. During osseointegration, the interfacial zone between the living bone and the titanium/titanium alloy dental implant materials, between 21 and 50 nm, plays a vital role since bone cells release critical growth factors into this interfacial zone for bone formation around the titanium dental implants. Furthermore, blood plasma proteins are deposited onto the surface oxide layer found on the surfaces of titanium dental implants after implantation, leading to the development of fibrin matrices, which act as a scaffold for the bone-forming cells to reside and therefore promote bone formation to anchor the dental implants [23,24].

One example of a titanium dental implant is the OsseoSpeed implant (DENTSPLY Implants, Mannheim, Germany), which came into the market in 2004. This implant’s surface texture results from two subtractive, sequential manufacturing steps: titanium oxide blasting and subsequent hydrofluoric etching. Titanium oxide blasting produces the microscale surface roughness, and subsequent etching with the hydrofluoric acid influences the nanostructure of the implant [25,26,27]. Ellingsen et al. have examined the biomechanical features and histomorphometric characteristics of osseointegration with OsseoSpeed implants using a rabbit model. For the treatment group that received OsseoSpeed implants, significantly greater removal torque and shear strengths and higher levels of bone to implant contact were observed after 1 and 3 months of healing compared to the controls [28,29,30]. In a healing chamber model, the amount of bone formation around OsseoSpeed implants was superior to the bone quantity around the precursor implant. Moreover, Choi et al. compared OsseoSpeed implants with TiUnite implants in a rabbit model and noted similar findings of osseointegration [31]. In prospective clinical trials, Mertens and Sterling examined the long-term clinical outcome of OsseoSpeed implants by evaluating 42 implants in 15 patients over five years. The overall survival rate was 97%, and the mean marginal bone loss was 0.1 mm. Such results seemed independent of immediate or conventional loading. In addition, Raes et al. reported a one-year survival rate of 98% in a prospective clinical trial using immediately professionalized OsseoSpeed implants placed in the anterior maxilla of 48 patients [32]. A 2-year prospective clinical trial by Collaert et al. examined the clinical outcomes of 25 edentulous patients; each was treated with five OsseoSpeed mandibular implants professionalized with the loaded screw-retained restoration. In this study, the two-year survival rate was 100%, and the mean crestal bone loss was measured as 0.11 mm [33].

### 4.2. Titanium Alloy

Despite the successful application of titanium implants, research has constantly aimed to develop advanced titanium alloying techniques to optimize biocompatibility and mechanical properties. However, Ti implants usually cannot be placed in narrow bones such as the anterior alveolar ridge [34]. In addition, close proximity between the implant and neighboring teeth could cause bone loss [35]. Thus, different titanium alloys have been developed to improve the mechanical strength for applications requiring small-diameter implants (≤3.5 mm) [36]. Titanium–6aluminum–4vanadium is one of the most commonly used titanium alloys. Ti alloy’s most commonly used product in dental implants is Ti–6Al–4V, known as Grade V titanium alloy, composed of 6 and 4% aluminum and vanadium with the addition of a maximum of 0.25% of iron and 0.2% of oxygen. Ti–6Al–4V yields better strength and fatigue features, excellent corrosion resistance, and an improved elastic modulus compared to cp-Ti. Specifically, vanadium has been demonstrated with high cytotoxicity, and aluminum might play a role in inducing senile dementia [37]. However, a safety risk is posed due to the release of toxic vanadium and aluminum ions [38,39]. Titanium–nickel is also limited due to nickel hypersensitivity [40].

In comparison, titanium alloys with other beta-phase stabilizers such as tantalum, molybdenum, niobium, and zirconium are non-toxic and non-allergenic and thus have received more interest as materials for medical applications [41,42]. Zirconium has the same crystal structure as Ti and exhibits unlimited mutual solubility in Ti [43]. Titanium–zirconium alloys (TiZr) have demonstrated increased corrosion resistance [44], improved tensile and fatigue strength [45,46], and similar biocompatibility as Ti [36,47,48]; as titanium and zirconium are the only metals that do not show osteoblast growth inhibition, a combination of both is well suited for implants [18]. TiZr alloy, known as Roxolild^®^, Straumann AG (Basel, Switzerland), contains 13 to 17% zirconium. Its surfaces are pretreated with large-grit (0.25–0.5 mm) aluminum oxide sand blasting and acid etching in hydrochloric and sulfuric acid [49]. In this context, Gottlow et al. could show significantly higher removal torque and bone area in vivo for a titanium–zirconium alloy compared to commercial pure (cp) titanium [50].

Furthermore, it was observed that the oxides on titanium–zirconium alloy surfaces are more stable and have favorable corrosion resistance [51]. Moreover, the alloying of titanium with zirconium improves the mechanical strength, especially for applications in small-diameter implants [36]. While the mechanical strength is high for titanium–zirconium alloys, they are well suited for implantation in the cortical bone due to a low Young’s modulus, which prevents stress shielding [52]. The effect of Zr on the increase in mechanical properties and its ability to influence the etching process were identified as causes for these differences [53]. Increased mechanical properties were responsible for fewer structural changes on TiZr during sand blasting [49]. TiZr increased integrin-beta3 mRNA and protein levels compared with Ti in an in vitro study by Gomez et al. Cells on TiZr surfaces showed higher MMP1 protein levels than Ti surfaces, although similar TIMP1 protein production was observed [54], suggesting that TiZr is a potential clinical candidate for soft tissue integration [55,56].

Furthermore, the alloying of zirconium was reported to influence the corrosion resistance of titanium alloys and acted as a catalytic agent for the formation of hydrogen during etching and hydridation [51,53]. In addition, the mechanical properties of titanium–zirconium alloys allow the placement of small-diameter implants in critical implantation sites, such as the front of the lower jaw, where bone is scarce and the crestal bone is thick [57]. An alternative alloy could consist of Ti, Ta, Nb, and Zr, which showed similar cytocompatibility to cpTi, but with a lower inflammatory response, and also osseointegrated [58], e.g., Ti–Ta–Nb–Zr(–Si)(–Fe) displayed improved cytotoxicity when compared to Ti–6Al–4V alloy [59].

Even though the side effects of these components have not been observed when they are used in the format of Ti alloy as dental implants, extra caution should be taken, and long-term evaluations should be conducted for safety concerns. Animal studies have shown the superior mechanical properties of titanium alloy compared with titanium alone when used as an implant material for a tooth implant. Biological responses to the alloys have been characterized in vitro [60,61,62]. It has been noted that the form of alloy has beneficial influences on its microstructure and, as a result, its mechanical properties. Randomized, controlled clinical trials on alloying with titanium are still scarce. A review of the available studies by Wennerberg et al. noted little clinical evidence so far to demonstrate a preference of alloying with titanium over zirconia or titanium alone. In a split-mouth study, alloying with titanium was compared with titanium alone, with early loading protocols in irradiated patients. One hundred and two implants were placed in 20 patients in both jaws. One-year follow-up showed excellent yield strength and fatigue properties for all implants, which translated to higher survival rates and low crystal bone loss <0.4 mm in all patients, with no significant difference. Accordingly, alloying with titanium was found to have low wear resistance, a high elastic modulus approximately 4–10 times that of human bone, and less shear strength, which could impair the usage as implants and in screw form.

### 4.3. Zirconia

Zirconia-based dental bioceramics are chemically inert materials with no adverse effects on oral tissues [63]. Zirconia can exist in several different crystal structures; however, the three molar percentage yttrium-stabilized tetragonal zirconia polycrystal (3Y-TZP) is the most commonly used for dental implants [64]. Zirconia has been increasingly used in dental implantology because of its ideal physical, aesthetic, and biological properties [64]. One of the selling points for manufacturers of zirconia implants is that its white color has advantages over metallic implants in narrow ridges. Zirconia, being white in color, avoids “black line” for Ti dental implants in patients with gingival and bone recession [65]. Unlike titanium, zirconia is bioceramic, which offers superior biological and anti-corrosive properties but also makes it more brittle. Zirconia implants are found to have higher survival and marginal bone loss than titanium dental implants after ten years or more from implantation [66]. Moreover, zirconia implant material has shown considerably higher cell spreading and cell viability and improved biocompatibility over titanium [64]. The other advantage of using zirconia is its high corrosion resistance, low infection rate, and plaque formation. Increasing success and survival rates and high biocompatibility make zirconia an ideal dental implant material candidate [67].

In a recent prospective cohort study, Balmer et al. evaluated a single zirconia implant’s radiological and clinical results with fixed dental prostheses or restored with single crowns for 60 months. Seventy-one zirconia implants were placed on the 61 patients’ posterior, anterior, and sites, and in a 60-month follow-up, the results indicated that the zirconia dental implants had a mean bone loss of 0.70 ± 0.60 mm after 60 months [68]. The authors also found that zirconia dental implants had a survival rate of approximately 98.40% (95.0% C.I. = 91.6, 99.90). Furthermore, the statistical analysis revealed no significant marginal bone level after the 60 months (*p* = 0.458), implying that zirconia dental implants had a lower/marginal bone level [68]. Therefore, it was concluded that zirconia implant material has mucosal margin levels, highly stable marginal bone, and higher survival rates. Moreover, it may serve as a reliable and safer implant material for dental implant applications.

## 5. Implant Surface Modifications Prevent Inflammation

### 5.1. Improvement of Tissue Integration on Implant

Various surface treatments have been commonly applied to improve the surface properties of dental implants. These physiochemical modifications can change the dental implants’ surface topography, morphology, and chemistry. Furthermore, additive processes are known to improve the physiological reaction of implants [33] potentially.

Morphology and topographical surface modifications can improve the interaction between implant and tissue [69,70]. In this context, rough implant surfaces promote osseointegration more than smooth surfaces [26,71]. Mechanical or chemical methods, or a combination of both, can be used to optimize the surface morphology and topography [72,73]. For example, one recognized method to modify titanium implant surface roughness is through blasting with titanium dioxide (TiO_2_) particles, and the resulting roughness can be controlled via the mesh size of the blasting particles. It has been found previously that the optimal surface roughness for titanium dental implants lies in the range of 0.3–2.2 µm, which is the surface roughness range of commercial dental implants [74]. This would result in the highest improvement in bioactivity. Besides blasting, dental implant surfaces may be classified into different groups, according to surface roughness: (1) smooth implant surfaces with Sa < 0.5 μm; (2) minimally rough surfaces with Sa values between 0.5 and 1.0 μm; (3) moderately rough surfaces with Sa values between 1.0 and 2.0 μm; and (4), rough surfaces with Sa values > 2.0 μm [75]. Smooth surfaces are used clinically. Machined Brånemark, Osseotite, and Nanotite implants are examples of minimally rough implants. Moderately rough implants include SLA, TiUnite, OsseoSpeed, TiOblast, and the Southern Implants, whereas IMZ, TPS, Ankylos, Friadent, and Xive represent rough surfaces [74]. Etching and multistep etching are frequently used to roughen the surface of titanium dental implants [76,77,78,79].

Implant surface roughness alteration involves mechanical modification of the surfaces to improve the integration of abutment into the soft tissues. Various mechanical processes and treatments have been tested to alter the surface roughness of implant materials. These mechanical processes/treatments, such as machining, grinding, polishing, and blasting, improve the adhesion and clean the surfaces of dental implant materials simultaneously [80,81].

### 5.2. Implant Surface Chemistry Alteration

Nevertheless, hardly any modification of the surface morphology can be done without inducing changes in the chemical surface composition and vice versa. The etching processes used on titanium for surface modifications increase the amount of hydrogen on the Ti surfaces, as liberated hydrogen ions are attached to titanium’s outer surface layer in the form of titanium hydride [69,82,83]. This process can be influenced by the molar strength of the acid and the etching time. Several studies suggest that the hydrogen content induces faster healing and better osseointegration. Therefore, cathodic polarization (hydridation) was applied to increase titanium hydride’s layer thickness and concentration [84]. Videm et al. showed that hydride surfaces with increased hydrogen content had 60% higher retention in an in vivo model [69]. In addition, the hydridation process can be used to improve the attachment of ampholytic biological molecules, which bind to the surface during hydridation together with hydrogen [84].

As the oxide layer on titanium is the most prominent material feature, modifications of this layer were tested. Nevertheless, the approaches to increase titanium’s biocompatibility by the sheer increase in oxide layer thickness by anodic oxidation (hydroxylation) in acidic solutions did not show increased biocompatibility [85,86,87]. Nevertheless, if hydroxylation is used with alkaline solutions, an increase in hydroxide (OH) groups on the surface can be achieved [88,89].

Alteration of the implant surface chemistry involves various chemical processes to achieve higher physical and mechanical properties. As a result, chemistry alteration of the implant surface will lead to improved performance of the dental implant material, and improved survival and success rates of the dental implants for several years [90]. Chemical treatments used for the surface chemistry alteration of dental implant materials’ surfaces can be categorized into acid treatment, alkali treatment, and the use of hydrogen peroxide and anodic oxidation. For example, anodic oxidation aims to increase the thickness of titanium (IV) oxide on the surfaces of dental implant materials. Similarly, hydrogen peroxide adds a porous outer layer and dense inner oxide layer on the surfaces of dental implant materials to improve the corrosion resistance features of the surfaces of the dental implant materials. On the other hand, alkali and acid treatments used in implant surface chemistry alteration focus on improving the biocompatibility features of the dental implant materials (Nicholson, 2020). In the following section, the surface chemistry alteration of titanium and titanium alloy dental implant materials will be discussed as titanium is most widely used in dental implants.

**Figure 1:**
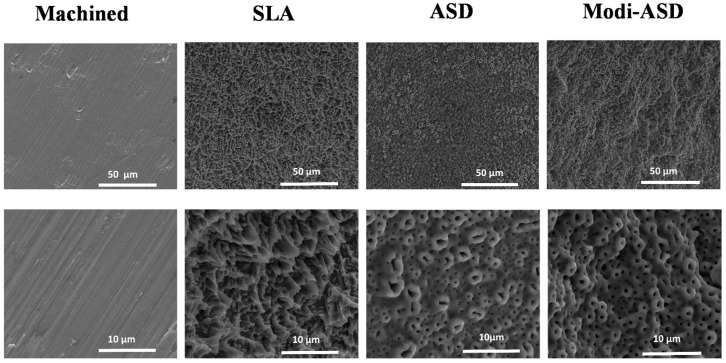
Scanning electron microscopy (SEM) images of surface-modified titanium substrates. SLA: sand-blasted and acid-etched Ti, ASD: anodized Ti, Modi-ASD: sand-blasted/acid-etched and anodized Ti. Reprinted with permission from licensee MDPI, Basel, Switzerland, under the terms and conditions of the Creative Commons Attribution license CC BY 4.0 [93].

Surface modification of titanium and titanium alloy dental implant materials, such as Ti–6A1–4V and cpTi (commercially pure titanium), is performed through the oxidization of titanium (IV) [91] (Figure 1). Changes in the dental material surface significantly promote the adhesion of osteoblasts and the oxide layer, which further enhances their biological properties, making them suitable for dental implantology applications [22]. Nevertheless, this implant surface chemistry alteration could potentially induce an immune response and develop fibrosis in the region surrounding the dental implants because the body more easily recognizes the chemically modified surface as an invader, and the release of various fibrotic factors will occur [92].

## 6. Biomolecules

Besides physical and chemical modifications to the implant surface, various bioactive molecules are also developed to treat the implant surface to increase biocompatibility [94,95]. By conjugating bioactive molecules, such as proteins, enzymes, or peptides, to the implant surface [95], the goal is to mitigate the host response that the implant will otherwise elicit after surgery and improve the interaction between the implant and cells at the implant site. Cell attachment factors such as fibronectin could improve the attachment and spread of cells on the implant surfaces, while the application of growth factors such as bone morphogenetic proteins could directly influence the development of osteoblasts on the surfaces [96]. Theoretically, the possibilities of suitable molecules are extensive, yet the process is limited by the conditions that molecules are exposed to during the coating and solubility and degradation problems or simply destruction due to the attachment process. Furthermore, polarization of the molecule (ampholyte) can be used for attachment to titanium in an electrochemical process. For example, two biomolecules, doxycycline and simvastatin, as coating candidates with such a technique have been reported [97,98,99,100,101].

**Figure 2:**
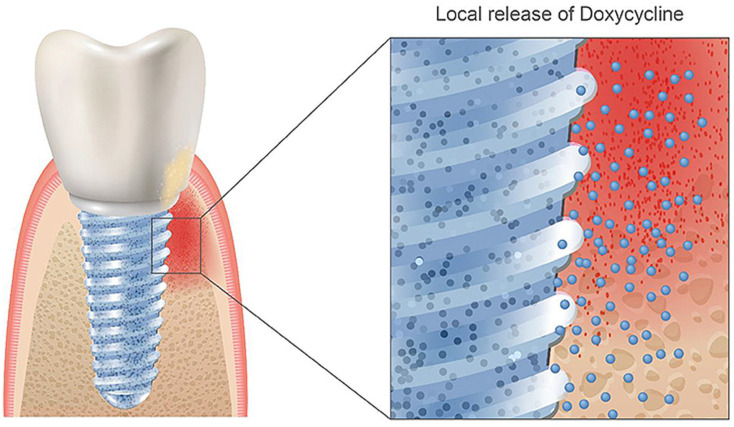
Coating a doxycycline layer on TiZr implants could be favorable for reducing or removing the antibiotics via oral administration after the implantation surgery. Reprinted with permission from Creative Commons License (CC BY 4.0) [99].

Doxycycline is a semi-synthetic broad-spectrum antibiotic from the group of tetracycline antibiotics, which is used to treat various infections and works by inhibiting bacterial protein biosynthesis [102,103]. Tetracycline enhances bone formation, mostly based on general knowledge about the interaction with collagen formation and calcium incorporation [104]. It has been proven to promote bone growth and treat periodontal disease and peri-implantitis in vitro [105,106,107]. Other studies further verified doxycycline’s applications in controlling osteogenic differentiation in genetically engineered mesenchymal stem cells [108]. Currently, doxycycline is mainly applied alone through drug delivery systems for periodontal disease and peri-implantitis treatment [107,109,110,111]. Combining doxycycline with dental implants and its direct incorporation in the implant system could be favorable as local administration would reduce interference with the patient’s body and optimize the area of drug administration to the bone directly surrounding the implant (Figure 2). However, successful binding of doxycycline directly to an implant has not been reported in the literature yet, but this could be achieved with the process of cathodic reduction in acidic electrolytes. However, one must be aware of the delicate dose relation between enhancing and inhibitory effects on the osteogenic differentiation of this biomolecule [112].

Statins benefit various medical conditions and are commonly used as cholesterol-lowering drugs. Statins have also been researched for their tumor inhibition potential and are used as anti-inflammatory drugs [113,114,115]. Moreover, the applications of statins could be further broadened to bone growth promotion [113,116,117,118]. Many studies have pointed out the capability of statins to reduce bone resorption by inhibiting osteoclast activity, which is essential for osteoporosis treatment [115,117,119,120,121]. Mechanistically, statins contribute to bone formation and stimulate bone growth by regulating bone morphogenetic protein-2 (BMP-2). In addition, the upregulation of BMP-2 causes increased osteoblast differentiation and bone formation, as documented in various works in the literature [113,117,118,122,123]. Another feature of statins is their ability to enter the cell membrane through passive diffusion and active uptake by osteoblasts [114,124,125].

It is worth noting that the surface modification of zirconia, including both physical and chemical modifications, is mainly similar to that of titanium and titanium alloy (including TiZr alloy). Specifically, sand blasting and acid etching are applied to increase roughness; various coating strategies use hydroxyapatite, calcium phosphate, electrophoretic deposition, and biofunctionalization with arginine–glycine–aspartate (RGD) peptide, which are utilized to improve biocompatibility and reduce inflammation with zirconia dental implants [126,127,128].

## 7. Meta-Study Analysis

The initial article pool had 40 articles. Standard reviews were excluded due to the possibility of study selection bias, and in vitro studies were excluded due to their limited clinical relevance. Subsequently, 20 publications were subjected to additional evaluation, including six animal studies with dog and monkey models, four human studies, and 20 clinical studies. The clinical studies can be further categorized as follows: seven RCTs (level 1b), one prospective controlled (level 2a), seven prospective uncontrolled studies (level 2b), one case series, and four case reports (level 4). The extensive examination brought about the final sample of nine articles, including three animal studies, two human studies, and four RCTs. The meta-analysis results on all the implant materials on marginal bone loss (MBL) showed that bone level 30 studies showed an interproximal marginal bone loss. The mean bone loss differs from 0.2–0.4 mm to 1.05–1.48 mm from the zirconia implant and also 0.3–0.5 mm to 0.67–1.43 mm for the titanium implant. The distal and mesial marginal bone loss was reported in some of the articles. The meta-analysis was conducted to examine the same intervention and outcomes for the 30 included studies. The mean difference for the unceasing outcome (MBL) was used, using the software of a random effect model (RevMan 5.3, 2014).

### 7.1. Evaluation of Heterogeneity

The Cochran test examined any discrepancies in treatment effects’ estimation from different RCTs for heterogeneity, and the difference was considered significant if *p* < 0.1. Statistics from 30 studies describing the total difference across the trials were used to compute heterogeneity, and results above 50% were viewed as moderate to high heterogeneity. All the findings for the included studies were pooled using the random model effect as the statistical heterogeneity among the studies was significant (93%, *p* < 0.00001). The mean difference for marginal bone loss between zirconia and titanium implants for all the pooled findings was −0.20 (−0.32–0.08), with a 95% confidence level. The overall estimate was statistically significant, with *p* < 0.0009. The meta-analysis was conducted with the continuous outcome using the random effect model (Table 1).

### 7.2. On the Effect of Implant Materials on Probing Depth

Overall, 22 studies out of 30 recorded the pocket probing depth. Albornoz et al. measured the PPD at six sites, and the other eight papers measured it at four areas. After a one-year follow-up (112, 15), it was noted that the mean pocket depth for the titanium abutment was 3.3 mm and the mean pocket depth for all the ceramic zirconia implants ranged from 2.9 to 3.5 mm.

DeAlboroz et al. noted that after a year of follow-up, an increase of 0.2 mm from the baseline was recorded around the zirconia implant, while the pocket probing depth around the titanium implant was not affected. In recent years, the mean pocket depth around zirconia abutment was found to be 3.38 mm, and the mean pocket depth around titanium alloy was 3.33 mm. After six months of follow-up, the zirconia abutment showed a pocket probing depth of 3.2 mm versus 3.4 mm at the sites of the titanium abutment. The survival rate after two years was used by two studies. Zembic et al. indicated that the mean pocket probing depth around the zirconia implant was 3.3 mm, with an upsurge of 0.4 mm from the baseline, and the titanium abutment had 3.6 mm, with an upsurge of 0.5 mm from the baseline. Lops et al. showed 2.6 mm for zirconia abutment and 2.7 mm for titanium. All the studies included showed no significant differences between zirconia and titanium implants. The pocket probing depth mean difference used in this meta-analysis was −0.10 (−0.25–0.05) with a 95% confidence level. The overall evaluation was not statistically significant at *p* = 0.18. Therefore, the meta-analysis with the random effect model was performed for a continuous outcome [130] (Table 2).

Abrahamsson et al. performed a comparison of peri-implant tissues on titanium and gold alloys. In total, 32 titanium implants were placed in five dogs, and the distance from the abutment–implant junction to the first bone–implant contact was considered to indicate the actual bone loss. Histometric observations indicated that bone loss was 0.78 around the titanium (control implant), 0.80 mm around alloy, 1.80 mm around zirconium, and 1.26 mm around the dental porcelain implant. The clinical evaluation indicated marked soft tissue recession around the alloy implant [137]. According to Piattelli et al., there was a difference in peri-implant tissue stability between titanium abutment versus gold alloy, zirconia, and aluminum oxide implants [138]. The research was conducted through various methods, including examining databases, dental implants, prosthetics, and periodontal journals. The research showed that the measurement of soft tissue had a problem with accuracy; peri-implant tissues around zirconia and titanium were defined in histologic and animal studies only. As a result of the difference in research types, follow-up time, and outcome variables, it was not easy to perform the meta-analysis. For example, titanium abutment did not have higher bone level maintenance than the gold alloy, aluminum oxide, or zirconia abutment [139], and there was no information about the zirconia and alloy’s clinical performance compared to the titanium.

Implant-supported restorations require crystal bone stability and healthy soft tissues, and both factors should be considered to determine a practical treatment approach for patients. The peri-implant tissue has been challenged for a while by some problems. Studies show that bone loss has been observed during the treatment in the first year. Implant materials are often regarded as the factors that affect the stability of the mucosa and crestal bone. In addition, several papers show similar responses to peri-implant tissues’ reaction to titanium and aluminum oxide implants [140].

A comparison of peri-implant tissues’ reaction on titanium and alloy implants was studied in dogs. Bone loss was considered as the distance from implant–implant junction to first bone–implant contact. Through observation, bone loss was 0.78 mm around the titanium implant and 1.80 mm around the alloy implant [48]. Zirconia and titanium implants were compared by placing 12 implants on six monkeys. There was no difference observed between treatment groups that received either material implant. The ability to form stable peri-implant tissues was tested using one piece of alloy and titanium implants. The report showed a vertical extension of soft peri-implant tissues around implants from the mucosa’s margin to the first bone–implant contact [141].

### 7.3. Clinical Studies

A histological study of the soft tissue response to titanium and zirconium healing caps/abutments in five patients was carried out. After six months of healing, gingival biopsy specimens were derived from test and control implant sites. It revealed that inflammation prevailed for titanium specimens compared with zirconium. In addition, one piece of soft tissue in aluminum oxide and titanium implants was compared to twenty patients. The biopsies indicated the exact composition of peri-implant tissue among tested abutments [48].

A randomized trial with a split-mouth design was conducted over 4 years, comparing titanium and gold alloy restored with metal–ceramic crowns in 20 patients. Each patient was given two implants, one gold alloy and one titanium. Four years later, peri-implant tissues had no difference in response to gold alloy or titanium implant. In a clinical randomized controlled multicenter study, there was a comparison between aluminum oxide and titanium implants. Patients were given 34 test sintered aluminum oxide abutments containing 35 control implants and observed for one year. The following 15 patients were subjected to ten tests, and ten control abutment implants were followed up for three years. In the first group, bone loss was absent around the ceramic implant, and the second group had a loss of 0.3 mm after one year and 0.1 mm gain after three years [142]. A 5-year study was performed to observe the difference between ceramic and titanium. Thirty-two patients were given 103 implants. Fifty-three aluminum oxide ceramics were connected. Soft tissue around the implant and the teeth was healthy. Regarding bleeding of the peri-implant mucosa, there was no difference in ceramic and titanium implants. Less bone loss was observed with titanium abutment implants than ceramic implants [142].

## 8. Discussion

Clinical trials were designed correctly and employed randomization, but proper control groups were often omitted. Randomized controlled clinical studies provide reliable evidence but contain inherent drawbacks compared to other studies. There is a tendency to favor randomized trials and avoid lower-rank evidence. Therefore, it is essential to compare the results to those that failed to use inclusion criteria. This should not be considered a means to increase the review’s integrity but to determine whether there is a difference in included and excluded research results. The clinical format was regarded as the most reliable, while the animal studies were the least reliable.

Lops et al. reviewed implant material effects on peri-implant tissues, and no inclusion or exclusion criteria were used. The readers cannot rely on the authors’ subjective selection of the studies [132]. However, no clinical trials were accounted for, and the advice was based on in vitro and animal studies.

The formation of a stable peri-implant seal by a prosthetic implant material is categorized into two parameters: presence or absence of loss of bone and gingival recession. It was proven in an animal study that titanium and oxide ceramic implants could develop stable soft-tissue seals. Soft tissues adjacent to gold and porcelain-fused-to-metal implants indicated recession and crystal bone loss [134]. Another study showed no difference between soft and hard tissue integration around gold alloy and titanium one-piece implants. These two studies possess a methodological disparity. The first study used a two-piece implant, and the other used a custom-made piece implant. There was proof of implant disconnection, second-stage surgery with flap elevation, and soft tissue recession. Smooth tissue extension and bone apposition were not different among compared specimens. The study showed similar biocompatibility between zirconia and titanium.

The physiology between animals and humans is similar and forms the basis for animal studies, and the outcomes are relevant to humans but cannot be generalized to a clinical environment. Therefore, clinicians are left to rely on data collected from animals. However, simple case reports are more clinically valid than randomized animal experiments and are controlled well.

Studied animal data should be carefully interpreted if applied in the clinical environment, if the clinical evidence that is being relied on is unavailable. For example, evidence of gold alloy implants not maintaining stable peri-implant tissue relied on animal studies even when there were contradictory data from clinical reports. Therefore, the concept should be reassessed using clinical and histologic evidence.

Three published prospective randomized controlled clinical trials show stable soft and hard tissue around aluminum oxide implants. Loss of bone occurred, but there was no difference from the control titanium implant, in which biocompatibility had been proven long ago. All studies showed bone loss, but a data pool was impossible to achieve since the follow-up period ranged from one to five years. Oxide ceramic implants can achieve a stable marginal bone in a clinical situation. We can conclude that titanium and alloy have no crystal bone stability difference.

Unfortunately, there is no comparison between zirconia and titanium implants in a clinical trial. Therefore, a conclusion regarding the superiority or inferiority of zirconium over titanium is challenging. Some data can rely on tooth-controlled investigations. A study of four years provided clear information that shows that zirconia implants cause a favorable reaction in peri-implant tissues. Therefore, there should be a clinical trial to compare zirconia and titanium implants. All studies failed to include the exact gingival recession measurement. Clinical studies contained a report of observation of the status of peri-implant mucosa. Animals and histologic experiments may give insights into the structure and dimensions of soft tissues in contact with the different implants. Therefore, analyzing other studies is critical to understanding whether the implant material is essential for smooth tissue behavior.

One of this investigation’s goals was to assess the impact of various implant materials on bone changes, probing depth, plaque levels, and peri-implant mucosal inflammation. The authors, in their investigation, focused on the biological outcomes (pocket depth and plague levels). The authors aimed to exclude studies where the implant was compared to tooth bone restoration, apart from the implant. Hence, some studies with a follow-up of less than six months were omitted. This action can be considered appropriate; patient bias is avoidable by uncontrolled prospective clinical trials. Hence, the longest follow-up was three years. Generally, both implant materials’ results showed minor statistically significant differences. The evidence-based review examined the outcome for implant materials on bone loss. As per the visceral, human biology and various clinical studies, implant materials (zirconia and titanium) indicated no differences in bone stability. However, the current review does not show significant differences in pocket probing depths between the various implant materials. Further, it is essential to note that Agustín-Panadero et al. showed significantly lower pocket probing depth around the zirconium implant than the titanium implant [143].

This study showed a complete picture of zirconia and titanium implants [135]. New in vitro studies indicated that the surface roughness of various implant materials has a crucial role in the performance of cells for the implant materials. Zirconia surfaces offer better adhesion media for epithelial cells than titanium surfaces. It can be noted that the reduced pocket probing depth around the implant is strongly related to the adherence to the gingival cells. It is rather challenging to examine the impact of the implant material on plague accumulation due to implant materials not showing the oral cavity. The included articles overlooked biological or mechanical implications. The most notable was in one study [136]. It is important to note that a fistula triggered by the excel cement has been documented to be a factor causing biological complications. The findings were explained through the implant design. The superstructure margin is located subgingivally, approximately 1–1.5 mm below a gingival crest. The implant, supported by the fixed partial dentures, was cemented through dual-cured resin cement on the implant materials. Hence, due to biological complications, the removal of excess adhesive was challenging. Accordingly, the complete removal of excess resin cement is essential, even with a customized implant.

## 9. Conclusions

There is no clear evidence indicating that titanium is better than other implant materials. Clinical evidence suggests little difference between different implant materials in peri-implant bone stability. There is no difference in crystal bone loss statistically from studies. Animal histologic studies have the same peri-implant soft and hard tissue reaction to titanium and zirconium. There is an indication of a better response of human mucosa to zirconia implants than titanium. While evidence-based research does not offer a definitive decision on using ceramic or metallic implants for the alveolar bone response, some studies do not show better mechanical or biological performance for zirconia implants over titanium implants. The metal analysis showed a statistically significant advantage of zirconia implants over titanium regarding developing a favorable response to the alveolar bone.

## Figures and Tables

**Table 1:** Comparison for all implants. Outcome: marginal bone loss.

	All Implant Materials		
Study or Subgroup	Mean (mm)	Std. Dev. (mm)	Total
Al-Nawas [129]	−0.20	0.26	11
Altuna et al., 2016	−0.32	0.72	14
Apostu et al., 2017	−0.08	0.16	25
Balmer [68]	−0.015	3.65	20

**Table 2:** Comparison of all implants—ceramic zirconia versus titanium abutment. Outcome: pocket probing depth (mm).

Study	Zirconia			Titanium			Weight	IV, Random at 95% CI
	Mean (mm)	SD (mm)	Total	Mean (mm)	SD (mm)	Total		
De Alboroz et al.	0.3	0.2	20	0.4	0.3	14	11.9%	−0.39 (−0.43, −0.35)
Hossein [131]	0.6	0.07	11	0.61	0.12	15	12.8%	−0.03 (−0.1, −0.31)
Lops [132]	0.04	0.01	15	0.15	0.11	21	11.9%	−0.20 (−0.27, −0.13)
Nascimento [133]	0.1	0.56	6	0.45	0.61	22	11.0%	−0.33 (−0.61, −0.55)
Payer et al. [134]	0.05	0.16	9	0.31	0.20	13	12.8%	−1.03 (−1.44, −0.62)
Kumar et al. [135]	0.23	0.07	15	0.55	0.18	24	12.4%	−0.13 (−1.44, −0.62)
Zembic et al. [136]	0.15	0.25	16	0.23	0.32	11	10.6%	−0.06 (−0.71, −0.65)

Test for overall effect, 95% CI Z = 3.31 *p* = 0.0009.

## Data Availability

Data available upon requests to the corresponding author.

## References

[1] Epker B., Frost H. (1965). Correlation of bone resorption and formation with the physical behavior of loaded bone. J. Dent. Res..

[2] Berglundh T., Persson L., Klinge B. (2002). A systematic review of the incidence of biological and technical complications in implant dentistry reported in prospective longitudinal studies of at least 5 years. J. Clin. Periodontol..

[3] Hussain B., Haugen H.J., Aass A.M., Sanz M., Antonoglou G.N., Bouchard P., Bozic D., Eickholz P., Jepsen K., Jepsen S. (2021). Peri-Implant Health and the Knowing-Doing Gap—A Digital Survey on Procedures and Therapies. Front. Dent. Med..

[4] Mombelli A., Lang N.P. (1998). The diagnosis and treatment of peri-implantitis. Periodontol. 2000.

[5] Angkaew C., Serichetaphongse P., Krisdapong S., Dart M.M., Pimkhaokham A. (2017). Oral health-related quality of life and esthetic outcome in single anterior maxillary implants. Clin. Oral Implant. Res..

[6] Wennerberg A., Albrektsson T. (2010). On implant surfaces: A review of current knowledge and opinions. Int. J. Oral Maxillofac. Implant..

[7] Hussain B., Karaca E.O., Kuru B.E., Gursoy H., Haugen H.J., Wohlfahrt J.C. (2021). Treatment of residual pockets using an oscillating chitosan device versus regular curettes alone-A randomized, feasibility parallel-arm clinical trial. J. Periodontol..

[8] Mombelli A., Marxer M., Gaberthuel T., Grunder U., Lang N.P. (1995). The microbiota of osseointegrated implants in patients with a history of periodontal disease. J. Clin. Periodontol..

[9] Pontoriero R., Tonelli M.P., Carnevale G., Mombelli A., Nyman S.R., Lang N.P. (1994). Experimentally induced peri-implant mucositis. A clinical study in humans. Clin. Oral Implant. Res..

[10] Berglundh T., Armitage G., Araujo M.G., Avila-Ortiz G., Blanco J., Camargo P.M., Chen S., Cochran D., Derks J., Figuero E. (2018). Peri-implant diseases and conditions: Consensus report of workgroup 4 of the 2017 World Workshop on the Classification of Periodontal and Peri-Implant Diseases and Conditions. J. Clin. Periodontol..

[11] Figuero E., Graziani F., Sanz I., Herrera D., Sanz M. (2014). Management of peri-implant mucositis and peri-implantitis. Periodontol. 2000.

[12] Chala M., Anagnostaki E., Mylona V., Chalas A., Parker S., Lynch E. (2020). Adjunctive Use of Lasers in Peri-Implant Mucositis and Peri-Implantitis Treatment: A Systematic Review. Dent. J..

[13] Hong D.G.K., Oh J.H. (2017). Recent advances in dental implants. Maxillofac. Plast Reconstr. Surg..

[14] Leventhal G.S. (1951). Titanium, a metal for surgery. J. Bone Joint Surg. Am..

[15] Albrektsson T., Hansson H.A., Ivarsson B. (1985). Interface analysis of titanium and zirconium bone implants. Biomaterials.

[16] Albrektsson T., Brånemark P.I., Hansson H.A., Lindström J. (1981). Osseointegrated titanium implants: Requirements for ensuring a long-lasting, direct bone-to-implant anchorage in man. Acta Orthop..

[17] Wintermantel E., Ha S. (2009). Medizintechnik Life Science Engineering.

[18] Steinemann S. (1998). Titanium-the material of choice?. Periodontol. 2000.

[19] Kasemo B. (1983). Biocompatibility of titanium implants: Surface science aspects. J. Prosthet. Dent..

[20] Kasemo B., Lausmaa J. (1994). Material-tissue interfaces: The role of surface properties and processes. Environ. Health Perspect..

[21] Vora H.D., Shanker Rajamure R., Dahotre S.N., Ho Y.H., Banerjee R., Dahotre N.B. (2014). Integrated experimental and theoretical approach for corrosion and wear evaluation of laser surface nitrided, Ti-6Al-4V biomaterial in physiological solution. J. Mech. Behav. Biomed. Mater..

[22] Nicholson J.W. (2002). The Chemistry of Medical and Dental Materials (RSC Materials Monographs).

[23] Apostu D., Lucaciu O., Lucaciu G.D., Crisan B., Crisan L., Baciut M., Onisor F., Baciut G., Campian R.S., Bran S. (2017). Systemic drugs that influence titanium implant osseointegration. Drug Metab. Rev..

[24] Mavrogenis A.F., Dimitriou R., Parvizi J., Babis G.C. (2009). Biology of implant osseointegration. J. Musculoskelet. Neuronal Interact.

[25] Ellingsen J.E., Thomsen P., Lyngstadaas S.P. (2006). Advances in dental implant materials and tissue regeneration. Periodontol. 2000.

[26] Lamolle S.F., Monjo M., Lyngstadaas S.P., Ellingsen J.E., Haugen H.J. (2009). Titanium implant surface modification by cathodic reduction in hydrofluoric acid: Surface characterization and in vivo performance. J. Biomed. Mater. Res. A.

[27] Lamolle S.F., Monjo M., Rubert M., Haugen H.J., Lyngstadaas S.P., Ellingsen J.E. (2009). The effect of hydrofluoric acid treatment of titanium surface on nanostructural and chemical changes and the growth of MC3T3-E1 cells. Biomaterials.

[28] Ronold H.J., Ellingsen J.E., Lyngstadaas S.P. (2003). Tensile force testing of optimized coin-shaped titanium implant attachment kinetics in the rabbit tibiae. J. Mater. Sci. Mater. Med..

[29] Rønold H.J., Lyngstadaas S.P., Ellingsen J.E. (2003). Analysing the optimal value for titanium implant roughness in bone attachment using a tensile test. Biomaterials.

[30] Ronold H.J., Ellingsen J.E., Lyngstadaas S.P. (2002). Bone bonding assessed by tensile testing of osseointegrating coin-shaped titanium implants. J. Dent. Res..

[31] Choi J.Y., Lee H.J., Jang J.U., Yeo I.S. (2012). Comparison between bioactive fluoride modified and bioinert anodically oxidized implant surfaces in early bone response using rabbit tibia model. Implant Dent..

[32] Raes F., Renckens L., Aps J., Cosyn J., De Bruyn H. (2013). Reliability of circumferential bone level assessment around single implants in healed ridges and extraction sockets using cone beam CT. Clin. Implant Dent. Relat. Res..

[33] Collaert B., Wijnen L., De Bruyn H. (2011). A 2-year prospective study on immediate loading with fluoride-modified implants in the edentulous mandible. Clin. Oral Implant. Res..

[34] Saulacic N., Bosshardt D.D., Bornstein M.M., Berner S., Buser D. (2012). Bone apposition to a titanium-zirconium alloy implant, as compared to two other titanium-containing implants. Eur. Cell Mater..

[35] Geurs N.C., Vassilopoulos P.J., Reddy M.S. (2010). Soft tissue considerations in implant site development. Oral Maxillofac. Surg. Clin. N. Am..

[36] Grandin H.M., Berner S., Dard M. (2012). A Review of Titanium Zirconium (TiZr) Alloys for Use in Endosseous Dental Implants. Materials.

[37] Forbes W.F., Gentleman J.F., Maxwell C.J. (1995). Concerning the role of aluminum in causing dementia. Exp. Gerontol..

[38] Domingo J.L. (2002). Vanadium and tungsten derivatives as antidiabetic agents. Biol. Trace Elem. Res..

[39] Boyce B., Byars J., McWilliams S., Mocan M., Elder H., Boyle I., Junor B. (1992). Histological and electron microprobe studies of mineralisation in aluminium-related osteomalacia. J. Clin. Pathol..

[40] Okazaki Y. (2002). Effect of friction on anodic polarization properties of metallic biomaterials. Biomaterials.

[41] Niinomi M., Hattori T., Morikawa K., Kasuga T., Suzuki A., Fukui H., Niwa S. (2002). Development of low rigidity beta-type titanium alloy for biomedical applications. Mater. Trans..

[42] Ye W., Mi X., Song X. (2012). Martensitic transformation of Ti-18Nb (at.%) alloy with zirconium. Rare Metals.

[43] Thibon I., Ansel D., Gloriant T. (2009). Interdiffusion in β-Ti–Zr binary alloys. J. Alloy Compd..

[44] Khan M.A., Williams R.L., Williams D.F. (1999). Conjoint corrosion and wear in titanium alloys. Biomaterials.

[45] Ho W.F., Chen W.K., Wu S.C., Hsu H.C. (2008). Structure, mechanical properties, and grindability of dental Ti-Zr alloys. J. Mater. Sci. Mater. Med..

[46] Kobayashi E., Matsumoto S., Doi H., Yoneyama T., Hamanaka H. (1995). Mechanical properties of the binary titanium-zirconium alloys and their potential for biomedical materials. J. Biomed. Mater. Res..

[47] Pae A., Lee H., Kim H.S., Kwon Y.D., Woo Y.H. (2009). Attachment and growth behaviour of human gingival fibroblasts on titanium and zirconia ceramic surfaces. Biomed. Mater..

[48] Linkevicius T., Apse P. (2008). Influence of abutment material on stability of peri-implant tissues: A systematic review. Int. J. Oral Maxillofac. Implant..

[49] Bernhard N., Berner S., De Wild M., Wieland M. (2009). The binary TiZr Alloy—A newly developed Ti alloy for use in dental implants. Forum Implantol..

[50] Gottlow J., Dard M., Kjellson F., Obrecht M., Sennerby L. (2010). Evaluation of a New Titanium-Zirconium Dental Implant: A Biomechanical and Histological Comparative Study in the Mini Pig. Clin. Implant Dent. Relat. Res..

[51] Ferreira E.A., Rocha-Filho R.C., Biaggio S.R., Bocchi N. (2010). Corrosion resistance of the Ti–50Zr at.% alloy after anodization in different acidic electrolytes. Corr. Sci..

[52] Wen C.E., Yamada Y., Hodgson P.D. (2006). Fabrication of novel TiZr alloy foams for biomedical applications. Mater. Sci. Eng. C.

[53] Frank M.J., Walter M.S., Lyngstadaas S.P., Wintermantel E., Haugen H.J. (2013). Hydrogen content in titanium and a titanium-zirconium alloy after acid etching. Mater. Sci. Eng. C Mater. Biol. Appl..

[54] Gomez-Florit M., Ramis J.M., Xing R., Taxt-Lamolle S., Haugen H.J., Lyngstadaas S.P., Monjo M. (2014). Differential response of human gingival fibroblasts to titanium- and titanium-zirconium-modified surfaces. J. Periodontal Res..

[55] Xing R., Salou L., Taxt-Lamolle S., Reseland J.E., Lyngstadaas S.P., Haugen H.J. (2014). Surface hydride on titanium by cathodic polarization promotes human gingival fibroblast growth. J. Biomed. Mater. Res. A.

[56] Xing R., Lyngstadaas S.P., Ellingsen J.E., Taxt-Lamolle S., Haugen H.J. (2015). The influence of surface nanoroughness, texture and chemistry of TiZr implant abutment on oral biofilm accumulation. Clin. Oral Implant. Res..

[57] Al-Nawas B., Brägger U., Meijer H.J.A., Naert I., Persson R., Perucchi A., Quirynen M., Raghoebar G.M., Reichert T.E., Romeo E. (2011). A Double-Blind Randomized Controlled Trial (RCT) of Titanium-13Zirconium versus Titanium Grade IV Small-Diameter Bone Level Implants in Edentulous Mandibles—Results from a 1-Year Observation Period. Clin. Implant Dent. Relat. Res..

[58] Stenlund P., Omar O., Brohede U., Norgren S., Norlindh B., Johansson A., Lausmaa J., Thomsen P., Palmquist A. (2015). Bone response to a novel Ti-Ta-Nb-Zr alloy. Acta BioMater..

[59] Kopova I., Strasky J., Harcuba P., Landa M., Janecek M., Bacakova L. (2016). Newly developed Ti-Nb-Zr-Ta-Si-Fe biomedical beta titanium alloys with increased strength and enhanced biocompatibility. Mater. Sci. Eng. C Mater. Biol. Appl..

[60] Mat-Baharin N.H., Razali M., Mohd-Said S., Syarif J., Muchtar A. (2020). Influence of alloying elements on cellular response and in-vitro corrosion behavior of titanium-molybdenum-chromium alloys for implant materials. J. Prosthodont. Res..

[61] Mello D.C.R., de Oliveira J.R., Cairo C.A.A., Ramos L.S.B., Vegian M., de Vasconcellos L.G.O., de Oliveira F.E., de Oliveira L.D., de Vasconcellos L.M.R. (2019). Titanium alloys: In vitro biological analyzes on biofilm formation, biocompatibility, cell differentiation to induce bone formation, and immunological response. J. Mater. Sci. Mater. Med..

[62] Liu X., Chen S., Tsoi J.K.H., Matinlinna J.P. (2017). Binary titanium alloys as dental implant materials-a review. Regen. BioMater..

[63] Ionescu R.N., Totan A.R., Imre M.M., Tancu A.M.C., Pantea M., Butucescu M., Farcasiu A.T. (2022). Prosthetic Materials Used for Implant-Supported Restorations and Their Biochemical Oral Interactions: A Narrative Review. Materials.

[64] Gautam C., Joyner J., Gautam A., Rao J., Vajtai R. (2016). Zirconia based dental ceramics: Structure, mechanical properties, biocompatibility and applications. Dalton Trans..

[65] Grech J., Antunes E. (2019). Zirconia in dental prosthetics: A literature review. J. Mater. Res. Technol..

[66] Lorusso F., Noumbissi S., Francesco I., Rapone B., Khater A.G.A., Scarano A. (2020). Scientific Trends in Clinical Research on Zirconia Dental Implants: A Bibliometric Review. Materials.

[67] Rohr N., Bergemann C., Nebe J.B., Fischer J. (2020). Crystal structure of zirconia affects osteoblast behavior. Dent. Mater..

[68] Balmer M., Spies B.C., Kohal R.J., Hammerle C.H., Vach K., Jung R.E. (2020). Zirconia implants restored with single crowns or fixed dental prostheses: 5-year results of a prospective cohort investigation. Clin. Oral Implant. Res..

[69] Videm K., Lamolle S., Monjo M., Ellingsen J.E., Lyngstadaas S.P., Haugen H.J. (2008). Hydride formation on titanium surfaces by cathodic polarization. Appl. Surf. Sci..

[70] Liu X., Chu P.K., Ding C. (2010). Surface nano-functionalization of biomaterials. Mater. Sci. Eng. R. Rep..

[71] Mustafa K., Wroblewski J., Lopez B., Wennerberg A., Hultenby K., Arvidson K. (2001). Determining optimal surface roughness of TiO_2_ blasted titanium implant material for attachment, proliferation and differentiation of cells derived from human mandibular alveolar bone. Clin. Oral Implant. Res..

[72] Buser D., Schenk R., Steinemann S., Fiorellini J., Fox C., Stich H. (1991). Influence of surface characteristics on bone integration of titanium implants. A histomorphometric study in miniature pigs. J. Biomed. Mater. Res..

[73] Conforto E., Aronsson B., Salito A., Crestou C., Caillard D. (2004). Rough surfaces of titanium and titanium alloys for implants and prostheses. Mater. Sci. Eng. C Mater. Biol. Appl..

[74] De Bruyn H., Christiaens V., Doornewaard R., Jacobsson M., Cosyn J., Jacquet W., Vervaeke S. (2017). Implant surface roughness and patient factors on long-term peri-implant bone loss. Periodontol. 2000.

[75] Albrektsson T., Wennerberg A. (2004). Oral implant surfaces: Part 1--review focusing on topographic and chemical properties of different surfaces and in vivo responses to them. Int. J. Prosthodont..

[76] Rønold H.J., Ellingsen J.E. (2002). Effect of micro-roughness produced by TiO_2_ blasting. Tensile testing of bone attachment by using coin-shaped implants. Biomaterials.

[77] Rønold H.J., Lyngstadaas S., Ellingsen J.E. (2003). A study on the effect of dual blasting with TiO_2_ on titanium implant surfaces on functional attachment in bone. J. Biomed. Mater. Res..

[78] Wen H.B., Liu Q., De Wijn J., De Groot K., Cui F. (1998). Preparation of bioactive microporous titanium surface by a new two-step chemical treatment. J. Mater. Sci. Mater. Med..

[79] Wen H., Wolke J., De Wijn J., Liu Q., Cui F., De Groot K. (1997). Fast precipitation of calcium phosphate layers on titanium induced by simple chemical treatments. Biomaterials.

[80] Quirynen M., Bollen C.M., Papaioannou W., Van Eldere J., van Steenberghe D. (1996). The influence of titanium abutment surface roughness on plaque accumulation and gingivitis: Short-term observations. Int. J. Oral Maxillofac. Implant..

[81] Bollen C.M., Papaioanno W., Van Eldere J., Schepers E., Quirynen M., van Steenberghe D. (1996). The influence of abutment surface roughness on plaque accumulation and peri-implant mucositis. Clin. Oral Implant. Res..

[82] Aronsson B.O., Hjörvarsson B., Frauchiger L., Taborelli M., Vallotton P.H., Descouts P. (2001). Hydrogen desorption from sand-blasted and acid-etched titanium surfaces after glow-discharge treatment. J. Biomed. Mater. Res..

[83] Taborelli M., Jobin M., Francois P., Vaudaux P., Tonetti M., Szmukler-moncler S., Simpson J., Descouts P. (1997). Influence of surface treatments developed for oral implants on the physical and biological properties of titanium.(I) Surface characterization. Clin. Oral Implant. Res..

[84] Ellingsen J.E., Videm K., Opsahl L., Rønold H.J. (2000). Implants with Modified Surfaces for Increased Biocompatibility and Method for Production Thereof.

[85] Choi J., Heo S., Koak J., Kim S., Lim Y., Kim S., Lee J. (2006). Biological responses of anodized titanium implants under different current voltages. J. Oral Rehabil..

[86] Park K., Heo S., Koak J., Kim S., Lee J., Kim S., Lim Y. (2007). Osseointegration of anodized titanium implants under different current voltages: A rabbit study. J. Oral Rehabil..

[87] Wang G., Cheng X. (2001). The preliminary study on the oxide film of pure titanium treated by anodic oxidation. Zhonghua Kou Qiang Ke Za Zhi.

[88] Sul Y.-T., Johansson C.B., Petronis S., Krozer A., Jeong Y., Wennerberg A., Albrektsson T. (2002). Characteristics of the surface oxides on turned and electrochemically oxidized pure titanium implants up to dielectric breakdown: The oxide thickness, micropore configurations, surface roughness, crystal structure and chemical composition. Biomaterials.

[89] Yang B., Uchida M., Kim H.M., Zhang X., Kokubo T. (2004). Preparation of bioactive titanium metal via anodic oxidation treatment. Biomaterials.

[90] Canullo L., Annunziata M., Pesce P., Tommasato G., Nastri L., Guida L. (2021). Influence of abutment material and modifications on peri-implant soft-tissue attachment: A systematic review and meta-analysis of histological animal studies. J. Prosthet. Dent..

[91] Effah E.A., Bianco P.D., Ducheyne P. (1995). Crystal structure of the surface oxide layer on titanium and its changes arising from immersion. J. Biomed. Mater. Res..

[92] Sittig C., Textor M., Spencer N.D., Wieland M., Vallotton P.H. (1999). Surface characterization of implant materials c.p. Ti, Ti-6Al-7Nb and Ti-6Al-4V with different pretreatments. J. Mater. Sci. Mater. Med..

[93] Kim M.H., Park K., Choi K.H., Kim S.H., Kim S.E., Jeong C.M., Huh J.B. (2015). Cell adhesion and in vivo osseointegration of sandblasted/acid etched/anodized dental implants. Int. J. Mol. Sci..

[94] Ellingsen J.E., Lyngstadaas S.P. (2008). Medical Prosthetic Devices Having Improved Biocompatibility. U.S. Patent.

[95] Puleo D., Nanci A. (1999). Understanding and controlling the bone–implant interface. Biomaterials.

[96] Liu X., Chu P.K., Ding C. (2004). Surface modification of titanium, titanium alloys, and related materials for biomedical applications. Mater. Sci. Eng. R. Rep..

[97] Geissler S., Tiainen H., Haugen H.J. (2016). Effect of cathodic polarization on coating doxycycline on titanium surfaces. Mater. Sci. Eng. C Mater. Biol. Appl..

[98] Walter M.S., Frank M.J., Satué M., Monjo M., Rønold H.J., Lyngstadaas S.P., Haugen H.J. (2014). Corrigendum to “Bioactive implant surface with electrochemically bound doxycycline promotes bone formation markers in vitro and in vivo” [Dental 30 (2) (2014) 200–214]. Dent. Mater..

[99] Rahmati M., Lyngstadaas S.P., Reseland J.E., Andersbakken I., Haugland H.S., Lopez-Pena M., Cantalapiedra A.G., Guzon Munoz F.M., Haugen H.J. (2020). Coating doxycycline on titanium-based implants: Two in vivo studies. Bioact. Mater..

[100] Walter M.S., Frank M.J., Rubert M., Monjo M., Lyngstadaas S.P., Haugen H.J. (2014). Simvastatin-activated implant surface promotes osteoblast differentiation in vitro. J. BioMater. Appl..

[101] Frank M.J., Walter M.S., Bucko M.M., Pamula E., Lyngstadaas S.P., Haugen H.J. (2013). Polarization of modified titanium and titanium–zirconium creates nano-structures while hydride formation is modulated. Appl. Surf. Sci..

[102] Injac R., Djordjevic-Milic V., Srdjenovic B. (2007). Thermostability testing and degradation profiles of doxycycline in bulk, tablets, and capsules by HPLC. J. Chromatogr. Sci..

[103] Cunha B.A., Sibley C.M., Ristuccia A.M. (1982). Doxycycline. Ther. Drug Monit..

[104] Guerra W., Silva I.R., Azevedo E.A., Monteiro A.R.S., Bucciarelli-Rodriguez M., Chartone-Souza E., Silveira J.N., Fontes A.P.S., Pereira-Maia E.C. (2006). Three new complexes of platinum (II) with doxycycline, oxytetracycline and chlortetracycline and their antimicrobial activity. J. Braz. Chem. Soc..

[105] Gomes P.S., Fernandes M.H. (2007). Effect of therapeutic levels of doxycycline and minocycline in the proliferation and differentiation of human bone marrow osteoblastic cells. Arch. Oral Biol..

[106] Tamimi F., Torres J., Bettini R., Ruggera F., Rueda C., López Ponce M., Lopez Cabarcos E. (2008). Doxycycline sustained release from brushite cements for the treatment of periodontal diseases. J. Biomed. Mater. Res. A.

[107] Büchter A., Meyer U., Kruse-Lösler B., Joos U., Kleinheinz J. (2004). Sustained release of doxycycline for the treatment of peri-implantitis: Randomised controlled trial. Br. J. Oral Maxillofac. Surg..

[108] Moutsatsos I.K., Turgeman G., Zhou S., Kurkalli B.G., Pelled G., Tzur L., Kelley P., Stumm N., Mi S., Müller R. (2001). Exogenously regulated stem cell-mediated gene therapy for bone regeneration. Mol. Ther..

[109] Mundargi R.C., Srirangarajan S., Agnihotri S.A., Patil S.A., Ravindra S., Setty S.B., Aminabhavi T.M. (2007). Development and evaluation of novel biodegradable microspheres based on poly(d,l-lactide-co-glycolide) and poly(ε-caprolactone) for controlled delivery of doxycycline in the treatment of human periodontal pocket: In vitro and in vivo studies. J. Control Release.

[110] Victor S.P., Kumar T.S.S. (2008). BCP ceramic microspheres as drug delivery carriers: Synthesis, characterisation and doxycycline release. J. Mater. Sci. Mater. Med..

[111] Ryder M.I., Pons B., Adams D., Beiswanger B., Blanco V., Bogle G., Donly K., Hallmon W., Hancock E.B., Hanes P. (1999). Effects of smoking on local delivery of controlled-release doxycycline as compared to scaling and root planing. J. Clin. Periodontol..

[112] Park J.B. (2011). Effects of doxycycline, minocycline, and tetracycline on cell proliferation, differentiation, and protein expression in osteoprecursor cells. J. Craniofac. Surg..

[113] Garrett I., Gutierrez G., Mundy G. (2001). Statins and bone formation. Curr. Pharm. Des..

[114] Liao J., Laufs U. (2005). Pleiotropic effects of statins. Annu. Rev. Pharmacol. Toxicol..

[115] Tandon V., Bano G., Khajuria V., Parihar A., Gupta S. (2005). Pleiotropic effects of statins. Indian J. Pharmacol..

[116] Monjo M., Rubert M., Wohlfahrt J.C., Rønold H.J., Ellingsen J.E., Lyngstadaas S.P. (2009). In Vivo Performance Of Absorbable Collagen Sponges With Rosuvastatin In Critical-Size Cortical Bone Defects. Acta BioMater..

[117] Mundy G., Garrett R., Harris S., Chan J., Chen D., Rossini G., Boyce B., Zhao M., Gutierrez G. (1999). Stimulation of bone formation in vitro and in rodents by statins. Science.

[118] Wong R., Rabie A. (2005). Histologic and ultrastructural study on statin graft in rabbit skulls. Int. J. Oral Maxillofac. Surg..

[119] Staal A., Frith J., French M., Swartz J., Güngör T., Harrity T., Tamasi J., ROGERS M., FEYEN J. (2003). The ability of statins to inhibit bone resorption is directly related to their inhibitory effect on HMG-CoA reductase activity. J. Bone Miner. Res..

[120] Uzzan B., Cohen R., Nicolas P., Cucherat M., Perret G. (2007). Effects of statins on bone mineral density: A meta-analysis of clinical studies. Bone.

[121] Kaji H., Kanatani M., Sugimoto T., Chihara K. (2005). Statins modulate the levels of osteoprotegerin/receptor activator of NFkappaB ligand mRNA in mouse bone-cell cultures. Horm. Metab. Res..

[122] Maeda T., Matsunuma A., Kurahashi I., Yanagawa T., Yoshida H., Horiuchi N. (2004). Induction of Osteoblast Differentiation Indices by Statins in MC3T3-E1 Cells. J. Cell Biochem..

[123] Ayukawa Y., Yasukawa E., Moriyama Y., Ogino Y., Wada H., Atsuta I., Koyano K. (2009). Local application of statin promotes bone repair through the suppression of osteoclasts and the enhancement of osteoblasts at bone-healing sites in rats. Oral Surg. Oral Med. Oral Pathol. Oral Radiol. Endod..

[124] Pagkalos J., Cha J.M., Kang Y., Heliotis M., Tsiridis E., Mantalaris A. (2010). Simvastatin induces osteogenic differentiation of murine embryonic stem cells. J. Bone Miner. Res..

[125] Monjo M., Rubert M., Ellingsen J.E., Lyngstadaas S.P. (2010). Rosuvastatin promotes osteoblast differentiation and regulates SLCO1A1 transporter gene expression in MC3T3-E1 cells. Cell Physiol. Biochem..

[126] Pardun K., Treccani L., Volkmann E., Li Destri G., Marletta G., Streckbein P., Heiss C., Rezwan K. (2015). Characterization of wet powder-sprayed zirconia/calcium phosphate coating for dental implants. Clin. Implant Dent. Relat. Res..

[127] Kohal R.J., Bachle M., Att W., Chaar S., Altmann B., Renz A., Butz F. (2013). Osteoblast and bone tissue response to surface modified zirconia and titanium implant materials. Dent. Mater..

[128] Sandhyarani M., Rameshbabu N., Venkateswarlu K. (2014). Fabrication, characterization and in-vitro evaluation of nanostructured zirconia/hydroxyapatite composite film on zirconium. Surf. Coat. Technol..

[129] Al-Nawas B., Domagala P., Fragola G., Freiberger P., Ortiz-Vigon A., Rousseau P., Tondela J. (2015). A Prospective Noninterventional Study to Evaluate Survival and Success of Reduced Diameter Implants Made From Titanium-Zirconium Alloy. J. Oral Implantol..

[130] Pjetursson B.E., Zarauz C., Strasding M., Sailer I., Zwahlen M., Zembic A. (2018). A systematic review of the influence of the implant-abutment connection on the clinical outcomes of ceramic and metal implant abutments supporting fixed implant reconstructions. Clin. Oral Implant. Res..

[131] Hosseini M., Worsaae N., Schiodt M., Gotfredsen K. (2013). A 3-year prospective study of implant-supported, single-tooth restorations of all-ceramic and metal-ceramic materials in patients with tooth agenesis. Clin. Oral Implant. Res..

[132] Lops D., Bressan E., Chiapasco M., Rossi A., Romeo E. (2013). Zirconia and titanium implant abutments for single-tooth implant prostheses after 5 years of function in posterior regions. Int. J. Oral Maxillofac. Implant..

[133] Souza J.C., Silva J.B., Aladim A., Carvalho O., Nascimento R.M., Silva F.S., Martinelli A.E., Henriques B. (2016). Effect of Zirconia and Alumina Fillers on the Microstructure and Mechanical Strength of Dental Glass Ionomer Cements. Open Dent. J..

[134] Payer M., Heschl A., Koller M., Arnetzl G., Lorenzoni M., Jakse N. (2015). All-ceramic restoration of zirconia two-piece implants--a randomized controlled clinical trial. Clin. Oral Implant. Res..

[135] Kumar Y., Jain V., Chauhan S.S., Bharate V., Koli D., Kumar M. (2017). Influence of different forms and materials (zirconia or titanium) of abutments in peri-implant soft-tissue healing using matrix metalloproteinase-8: A randomized pilot study. J. Prosthet. Dent..

[136] Zembic A., Bosch A., Jung R.E., Hammerle C.H., Sailer I. (2013). Five-year results of a randomized controlled clinical trial comparing zirconia and titanium abutments supporting single-implant crowns in canine and posterior regions. Clin. Oral Implant. Res..

[137] Abrahamsson I., Cardaropoli G. (2007). Peri-implant hard and soft tissue integration to dental implants made of titanium and gold. Clin. Oral Implant. Res..

[138] Piattelli A., Pontes A.E., Degidi M., Iezzi G. (2011). Histologic studies on osseointegration: Soft tissues response to implant surfaces and components. A review. Dent. Mater..

[139] Al-Hashedi A.A., Laurenti M., Benhamou V., Tamimi F. (2017). Decontamination of titanium implants using physical methods. Clin. Oral Implant. Res..

[140] Bösch A., Jung R.E., Sailer I., Goran B., Hämmerle C.H., Thoma D.S. (2018). Single-tooth replacement using dental implants supporting all-ceramic and metal-based reconstructions: Results at 18 months of loading. Int. J. Periodontics Restor. Dent..

[141] Kohal R.J., Weng D., Bachle M., Strub J.R. (2004). Loaded custom-made zirconia and titanium implants show similar osseointegration: An animal experiment. J. Periodontol..

[142] Andersson B., Glauser R., Maglione M., Taylor A. (2003). Ceramic implant abutments for short-span FPDs: A prospective 5-year multicenter study. Int. J. Prosthodont..

[143] Agustin-Panadero R., Bustamante-Hernandez N., Labaig-Rueda C., Fons-Font A., Fernandez-Estevan L., Sola-Ruiz M.F. (2019). Influence of Biologically Oriented Preparation Technique on Peri-Implant Tissues; Prospective Randomized Clinical Trial with Three-Year Follow-Up. Part II: Soft Tissues. J. Clin. Med..

